# Implementing an Open Source Electronic Health Record System in Kenyan Health Care Facilities: Case Study

**DOI:** 10.2196/medinform.8403

**Published:** 2018-04-18

**Authors:** Naomi Muinga, Steve Magare, Jonathan Monda, Onesmus Kamau, Stuart Houston, Hamish Fraser, John Powell, Mike English, Chris Paton

**Affiliations:** ^1^ KEMRI/Wellcome Trust Research Programme Nairobi Kenya; ^2^ e-Health and Systems Development Unit Ministry of Health Nairobi Kenya; ^3^ Vimak Company Limited Nairobi Kenya; ^4^ Brown Center for Biomedical Informatics Brown University Providence, RI United States; ^5^ Nuffield Department of of Primary Care Health Sciences University of Oxford Oxford United Kingdom; ^6^ Nuffield Department of Medicine University of Oxford Oxford United Kingdom; ^7^ Centre for Tropical Medicine and Global Health Nuffield Department of Medicine University of Oxford Oxford United Kingdom

**Keywords:** electronic health records, software, medical records, Kenya, open source

## Abstract

**Background:**

The Kenyan government, working with international partners and local organizations, has developed an eHealth strategy, specified standards, and guidelines for electronic health record adoption in public hospitals and implemented two major health information technology projects: District Health Information Software Version 2, for collating national health care indicators and a rollout of the KenyaEMR and International Quality Care Health Management Information Systems, for managing 600 HIV clinics across the country. Following these projects, a modified version of the Open Medical Record System electronic health record was specified and developed to fulfill the clinical and administrative requirements of health care facilities operated by devolved counties in Kenya and to automate the process of collating health care indicators and entering them into the District Health Information Software Version 2 system.

**Objective:**

We aimed to present a descriptive case study of the implementation of an open source electronic health record system in public health care facilities in Kenya.

**Methods:**

We conducted a landscape review of existing literature concerning eHealth policies and electronic health record development in Kenya. Following initial discussions with the Ministry of Health, the World Health Organization, and implementing partners, we conducted a series of visits to implementing sites to conduct semistructured individual interviews and group discussions with stakeholders to produce a historical case study of the implementation.

**Results:**

This case study describes how consultants based in Kenya, working with developers in India and project stakeholders, implemented the new system into several public hospitals in a county in rural Kenya. The implementation process included upgrading the hospital information technology infrastructure, training users, and attempting to garner administrative and clinical buy-in for adoption of the system. The initial deployment was ultimately scaled back due to a complex mix of sociotechnical and administrative issues. Learning from these early challenges, the system is now being redesigned and prepared for deployment in 6 new counties across Kenya.

**Conclusions:**

Implementing electronic health record systems is a challenging process in high-income settings. In low-income settings, such as Kenya, open source software may offer some respite from the high costs of software licensing, but the familiar challenges of clinical and administration buy-in, the need to adequately train users, and the need for the provision of ongoing technical support are common across the North-South divide. Strategies such as creating local support teams, using local development resources, ensuring end user buy-in, and rolling out in smaller facilities before larger hospitals are being incorporated into the project. These are positive developments to help maintain momentum as the project continues. Further integration with existing open source communities could help ongoing development and implementations of the project. We hope this case study will provide some lessons and guidance for other challenging implementations of electronic health record systems as they continue across Africa.

## Introduction

### Background

A major driver of the increased use of electronic health record (EHR) systems in recent years has been the belief that these systems can support the provision of high-quality care [1,2]. Features such as a clinical decision support system can play a role in reducing medical errors by providing point-of-care information to support decision making by alerting a doctor to drug interactions when they create an electronic prescription [3]. More recently, EHRs have been proposed as the digital infrastructure to support learning health systems that enable continuous improvement through a cycle of EHR data analysis and quality improvement interventions [4-6].

In high-income countries, EHR adoption has been fostered by government incentive schemes such as the Health Information Technology for Economic and Clinical Health Act of 2009 in the United States through which health care providers have been compensated for the costs of information technology (IT) systems if they were able to demonstrate that the systems were used to improve care or increase efficiencies—so-called “Meaningful Use” [7,8].

Low-income countries, despite facing the challenges of resource constraints, inadequate data collection systems, the lack of incentives to collect health information, and inadequately trained personnel [9], have seen the increased use of EHR systems through aid-funded projects linked to specific diseases [1,10]. For example, in Kenya, EHRs have been used within projects that mainly support HIV care leading to well-developed systems for this disease area. For the management of both HIV and tuberculosis (TB), the result of digitization has been better record-keeping, patient management, follow-up, and stock control [11-14]. Although these implementations were largely successful, challenges encountered included limited interoperability with other systems and a lack of direct use by clinicians—systems are often used by clerks who enter data on behalf of the clinical team [15].

In the light of the perceived success of these disease-focused clinic-based systems, the Kenyan Ministry of Health (MOH) has begun to adapt one of the main systems (Open Medical Record System, OpenMRS) for use in public health facilities. This case study describes the current eHealth policies and guidance of the Kenya MOH and identifies the lessons learned from the initial development and implementation of this new OpenMRS-based system called Afya Electronic Health Management System (AfyaEHMS).

### Government eHealth Policy, Projects, and Guidance

#### Health Management Information Systems, Centers for Disease Control and Prevention, and National AIDS and STI Control Programme Electronic Medical Records Reviews (2007-2009)

Several assessments of systems used to manage patient and health data in Kenya (reporting systems and EHRs) were carried out between November 2007 and July 2009 by the Health Management Information Systems department (HMIS in MOH), the US Centers for Disease Control and Prevention (CDC), and the National AIDS and STIs Control Programme (NASCOP) [15]. The narrative synthesis of the findings of the 3 reviews highlighted a number of challenges encountered in previous EHR implementations. Specific challenges identified included varying data security functionality, unreliable vendor support, sustainability issues, variable reporting functionality, limited feedback for patient care, and limited ability to exchange information between systems [16]. Key benefits identified included HIV care systems that were highly developed and that were efficiently handling antiretroviral therapy care data.

From these assessments by HMIS, CDC, and NASCOP, recommendations were made regarding the way forward toward the scale up and harmonization of data systems for health services to improve patient care, facility and resource management, and policy development and evaluation [15].

#### Electronic Medical Records Standards and Guidelines Report (2010)

The recommendations from the HMIS, CDC, and NASCOP reviews then formed the basis of an “Electronic Medical Records Standards and Guidelines” (ESG) document for Kenya [17] in 2010. The aim of this document was to ensure quality of software, compatibility of data sharing, ease of maintenance, and common understanding among the workforce. The ESG document was designed to offer guidelines to the minimum standard for generic electronic systems in the health care setting for electronic medical record (EMR) system developers, implementers, and those contemplating the use of EMR systems. The guidelines covered the sections mentioned in Table 1.

**Table 1:** Sections covered in the Electronic Medical Records Standards and Guidelines (ESG) document. EMR: electronic medical record.

Section	Description	Target
EMR development	Outlines prerequisite processes of EMR development Identifies basic functional requirements for EMRs Identifies software attributes needed to ensure quality data and system security	EMR developers
EMR interoperability	Recommends that EMR systems can transmit and receive a minimum dataset via Health Level 7 messaging Recommends that systems have the capability to transmit aggregate data to District Health Information Software Version 2 via Statistical Data and Metadata eXchange for the Health Domain, in short SDMX.HD, messaging	EMR developers; program managers
EMR implementation	Outlines conditions to be met for successful EMR implementation	EMR implementers; program managers

#### Kenya Electronic Medical Records Review Toward Standardization (2011)

In 2011, a review of 17 EMR systems implemented in Kenya was carried out to assess the progress made toward standardization comparing the recommendations of the ESG document against the actual state of EMR use in health care facilities selected for review [16]. The review scored systems according to 7 functional areas: system details and standards; basic demographic and clinical health information; order entry and prescription; clinical decision support; health information and reporting; security and confidentiality; and exchange of electronic information. The results showed a wide variation of the capabilities of the different systems, variation in the adoption of functionalities of the same systems in different facilities, and variation in the overall adoption and use of systems across different facilities [16].

Of the systems reviewed, the EMR systems with the highest weighted scores over the 7 functional areas were OpenMRS AMPATH, IQ Care, and C-PAD at 95.2%, 90.3%, and 77.1%, respectively; these were systems used for HIV patient care [18].

#### Kenya National eHealth Strategy (2011-2017)

A Kenya National eHealth Strategy was developed in 2010, with an aim to harness information and communication technologies (ICT) for improved health care delivery by supporting informed policy, improving access to clinical evidence for care providers, fostering interoperability, and creating linkages between service providers and researchers [19]. The strategy outlines 5 key areas: telemedicine, health information systems, information for citizens, mHealth, and e-learning. The health information systems pillar was prioritized and divided into 5 functional domains: patient centric information; pharmacy and medical supply chain information; financial information; health workforce management; and training and regulation.

The strategy identified 6 principles that are key factors in its implementation: strong leadership and governance through a proposed National eHealth Steering committee; formation of partnerships for shared information and services among stakeholders; leveraging available resources (human, financial, and technical); safeguarding privacy and security; harmonization of disparate expertise (health and technological); phased implementation of prioritized initiatives; and ensuring redundancy in mission-critical aspects of eHealth systems.

#### District Health Information Software Version 2 (2011)

Kenya has adopted, at a national level, the District Health Information Software Version 2 (DHIS2) for aggregating health data across different levels of the health system. The DHIS2 system was implemented as a response to challenges with the previous Microsoft Excel file-based system. These included an inability to fully analyze the data collected due to the way the data were aggregated, a lack of error-checking capabilities, incomplete data, and limited capacity in the use of information for decision making [20]. DHIS2 offers several advantages: it is free and open source (licensed under the new Berkeley Software Distribution license), it allows for data collection and use at different levels of the health system, it has a Web-based interface allowing for access using several devices, and it has a good network of support from worldwide users [21]. Data are entered into the system by health records officers who are responsible for data management at the facility or county level. DHIS2 was implemented through the support of development partners and consultants from the University of Oslo, Norway, after extensive stakeholder consultations [22].

#### KenyaEMR (Open Medical Record System; 2012-2013)

KenyaEMR [23] is a tailored distribution of Open Medical Record System (OpenMRS), an open source EHR system that has been widely used in several African countries to support the management of HIV/AIDS patients (and more recently other diseases such as TB and noncommunicable diseases). OpenMRS was developed to provide a core system and range of plug-in modules from which clinical health information systems could be created to allow flexibility to include or exclude particular modules depending on the needs of the health care facilities where the software was to be installed [24]. The KenyaEMR system was designed to meet the requirements laid out in the ESG report and has now been implemented in over 300 facilities in 4 geographic regions in Kenya, with support from the International Training and Education Center for Health (ITECH Kenya) of the University of Washington [25]. ITECH Kenya also supports the use of the system through extensive capacity building through facility-based champion mentors.

**Table 2:** Summary of reports and projects deployed. HMIS: Health Management Information Systems; CDC: Centers for Disease Control and Prevention; NASCOP: National AIDS and STI Control Programme; EMR: electronic medical record; DHIS2: District Health Information Software Version 2; IQCare: International Quality Care; AfyaEHMS: Afya Electronic Health Management System.

Reports and projects	2007-2009	2010	2011	2012-2013	2014-2017
Reports	HMIS, CDC, and NASCOP EMR Evaluations	EMR Standards and Guidelines Report	EMR Review Toward Standardization; Kenya National eHealth Strategy (2011-2017)		
Deployments			DHIS2 Rollout	KenyaEMR Rollout; IQCare Rollout	AfyaEHMS Rollout

#### International Quality Care (2012-2013)

International Quality Care (IQCare) [26] is a freely available, Windows-based EHR application system that offers a variety of features for managing clinical care for primarily HIV or AIDS patients and has been deployed in over 300 facilities in Kenya. The system also has a supply chain management feature for management of drugs and other consumables. IQCare is implemented in Kenya through the support of the Palladium Group (formerly Futures Group) and is donor-funded through AIDS Relief. Palladium is an international consulting firm that works in various industries to provide customized solutions. In Kenya, they work closely with the MOH in a range of health areas including HIV and AIDS and more generally providing strategic information capacity building.

#### Afya Electronic Health Management System (2014-Present)

The challenges reported in the reviews and assessments coupled with the need to have a comprehensive picture of patient care from the lowest level of the health system to referral facilities led the MOH, supported by the World Health Organization (WHO), to commission the development of a county electronic health record (CEHR) system now called AfyaEHMS. Other partners supporting the project were Department for International Development (DFID) and United States Agency for International Development-funded projects AfyaInfo [27] and APHIAplus Northern Arid Lands [28]. It was envisioned that the system would be implemented in 2 counties: Turkana County (located in the Northern more sparsely populated areas of Kenya) which had relatively few existing implementations in public health facilities and in theory allowing for a faster county-wide scale up and Machakos County (located in the more central, semiarid but more developed part of Kenya) which already had a system in place but would provide a good comparison to the Turkana implementation. Table 2 shows a timeline of these reports and projects.

## Methods

### Overview

This case study has been developed in 2 phases over a period of 2 years. In the first phase, a research team from Kenya Medical Research Institute (KEMRI)/Wellcome Trust Research Programme (ME, JM, NM) supported by the University of Oxford (CP) was commissioned by the MoH and WHO to report on the initial plans and progress of the AfyaEHMS project. The second stage of this research followed the conclusion of the initial commissioned work and was undertaken as part of a new wider study investigating the use of open source software in public hospitals in Kenya bringing in coinvestigators from Leeds University (HF) and the Department of General Practice at the University of Oxford (JP). This new remit allowed the team to look at the AfyaEHMS project in the context of the wider health IT landscape in public hospitals in Kenya and to follow up the project as it proceeded to other counties beyond the initial implementation.

### Site Visits

To review the progress of implementation of the new system the team undertook 5 site visits to the county facilities (county referral hospital and health centers) over a period of 12 months to conduct semistructured and informal interviews with clinicians and IT staff. The first site visit to the county hospital was carried out to familiarize JM and NM with the site before system installation. Two site visits were done during installation of Version 1 of the system and 1 visit 6 weeks after the initial installation (JM). The last visit was carried out by NM after the developer had installed Version 2 of the system. During the site visits, informal interviews were carried out with the members of staff and field notes recorded as discussions took place. The team also attended 5 project meetings with the Kenyan implementation consultants and the MOH and 2 Skype calls with the developers and system users.

### Follow-Up

Following the conclusion of the initial consultancy, the research team conducted a series of informal discussions with MoH officials (eHealth Unit) and the implementing consultants (Vimak). The initial rollout of the AfyaEHMS project was scaled back and a new version developed and implemented in new counties across Kenya. The research team also had discussions with the new system developer on the progress of AfyaEHMS.

## Results

### System Specification and Requirements Gathering

**Figure 1:**
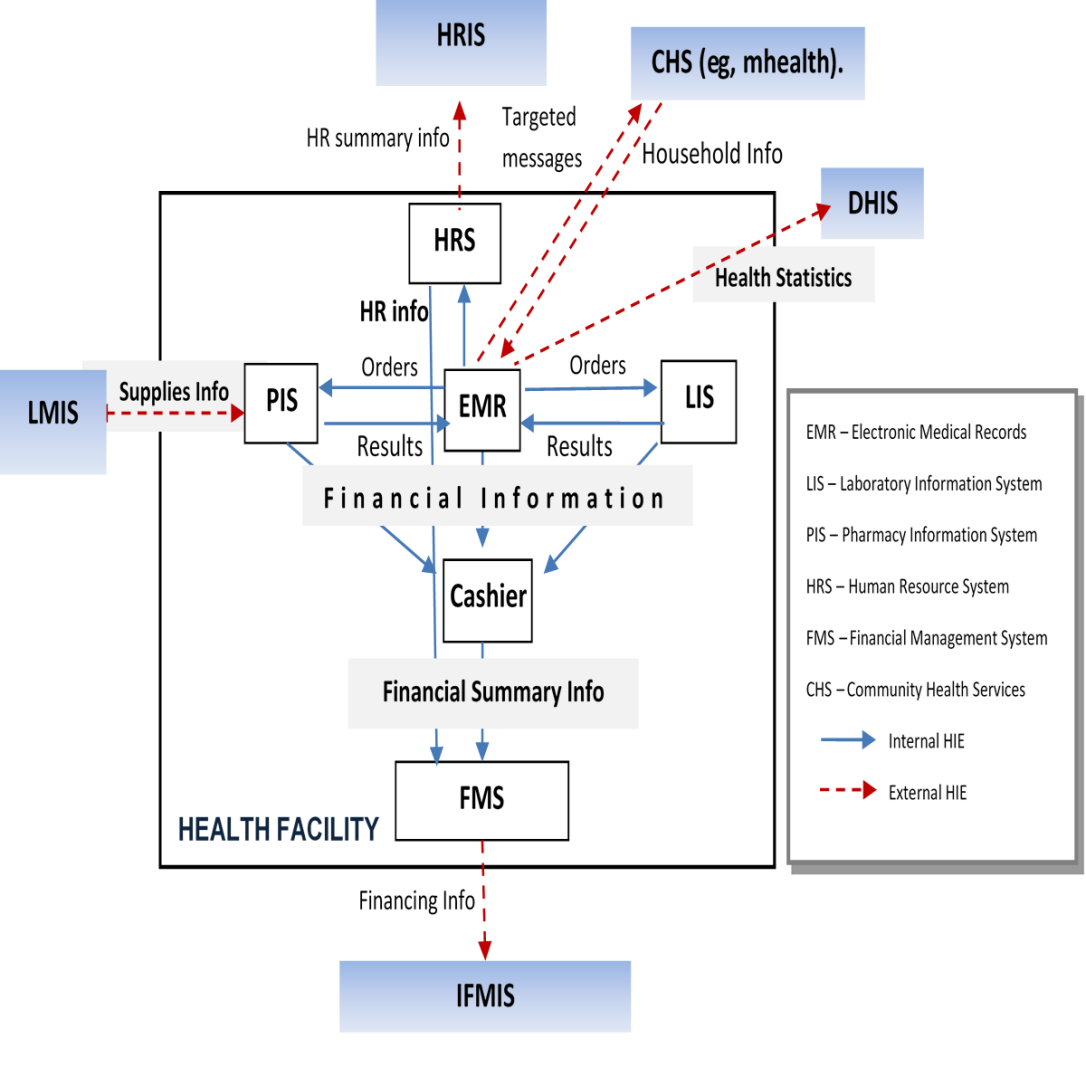
Proposed electronic health record at facility level (source: Kenyan Ministry of Health). LMIS: Logistics Management Information Systems; IFMIS: Integrated Financial Management Information Systems; DHIS: District Health Information Software; HRIS: Human Resources Information System; HIE: Health Information Exchange.

An MOH working group primarily concerned with carrying out monitoring and evaluation activities at the hospitals was charged with implementation of the CEHR. It was envisioned that the system would have a health information exchange (HIE) component to enable interoperability and sharing of data between the various modules of the EHR, within the hospital, between hospitals in the county, and into other health information repositories such as the national health information system (DHIS2) and the human resources information system. Information collected by the EHR would include management information such as financial and human resources, and individual electronic medical records including pharmacy and laboratory information. The system was envisaged to function with health workers entering primary data as part of their work or near real time through data clerks. Figure 1 shows the proposed EHR at facility level.

Working with WHO, MOH, and various stakeholders, the implementation consultants defined the EHR requirements and produced a specifications document outlining each component of the system (in-patients, laboratory, billing, etc) at the start of January 2013. The consultants met with hospital teams and, using structured forms, defined various use cases to be included in the new EHR. A use case definition included the use case description, definition of actors, triggers, conditions, normal and alternative flows, frequency of use, exceptions, dependent use cases, special requirements, assumptions, and other notes. The use cases were used to define modules that would be expected in the system including registration, outpatient, referrals, pharmacy, laboratory, inpatient, mother and child health (MCH), specialized clinics, billing, financial information management, human resources, logistics, HIE, and the community health system. A summary of the use case definition is outlined in Table 3.

**Table 3:** Electronic health record (EHR) use cases. Source: EHR validated use cases.

Use case ID	Primary actor (system users)	Use cases	Description
UC-1	Clerk, patient	Registration	Register patient into system to link the patient to other modules or facilities. Used for inpatients and outpatients
UC-2	Clinician, patient	Outpatient	Record clinical details of evaluation of patients
UC-3	Clinician	Referrals	Refer patients for tests, diagnosis, or treatment to internal department or external facility (specialist)
UC-4	Pharmacist, patient	Pharmacy	Receives prescription and dispenses drugs to patient; receive order from inpatient ward or other pharmacy within facility and manage bulk order
UC-5	Lab technician	Laboratory	Lab results or diagnosis recorded
UC-6	Clinician, patient	Inpatient	Admit patient to the ward for further management, discharge patients, patient referral to theater and handling of deaths
UC-7	Clinician, patient	Mother child health	Manage maternity, antenatal care, and immunization services
UC-8	Clinician, patient	Specialized clinics	Record clinical details of evaluation of patient
UC-9	Clerk, patient	Billing	Record charges for health service to patient, produce receipts
UC-10	Accountant or clerk	Financial information management	Record revenues and expenses for the facility
UC-11	HR office or administrator	Human resources	Record cadre workloads in facility
UC-12	Stores officer	Logistics	Receive or dispatch items into or out of store
UC-13	Various	Health information exchange	Return to point of service (PoS) unique patient ID from County Master Patient Registry; retrieve clinical data from another PoS application; push clinical data to electronic medical record for updating orders or prescriptions
UC-14	Community health worker	Community health system	Report vital event data (births, deaths) to County Civil Registration System using mHealth solutions

An assessment of the readiness of the target counties was also carried out to allow for proper planning and support of system rollout. In addition to the readiness assessment, a site visit to Machakos County was also conducted for the consultants and developers to understand the working of typical health facilities within a county. Consultations on the implementation of the system were also undertaken at the county level to engage the county leaders.

### System Selection and Development

The OpenMRS system was selected as the base platform for the implementation. A team of developers based in India were contracted to develop new system modules owing to prior experience in customizing OpenMRS for use in Indian hospital settings (in Kenya, OpenMRS had previously usually been implemented in small facilities such as HIV clinics).

The new EHR system would use the OpenMRS core architecture plus standard modules for patient management and clinical documentation. These modules would be augmented by an integrated suite of 10 modules for hospital management, including clinical, management, and administrative systems, customized specifically for workflow process within a district hospital system and integrated with DHIS2 using the WHO Statistical Data and Metadata eXchange for the Health Domain, in short, SDMX.HD standard.

### Implementation in Machakos County

The first county to implement the new system, now called AfyaEHMS, was Machakos County, which is the focus of this paper. The system was later also rolled out in Baringo County to primary care-level facilities (4 health centers and 1 dispensary) at the same time. Three of the facilities took up the system but gradually stopped using it due to issues with ongoing support for the system, and they were not able to wait until a newer version of the system was ready.

Machakos County has a population of slightly over a million people and has health facilities that can be grouped as district or mission hospitals, referral and provincial hospitals, health centers, dispensaries, private hospitals, private clinics, maternity hospitals and nursing homes, and special treatment centers with the referral hospital providing the highest level of care in the county and also serving as a referral facility for neighboring counties. Health service delivery in Kenya is a devolved function run by 47 counties. The health delivery system is classified into 4 levels of care with different facilities falling into the levels according to the services they provide [29] as summarized in Table 4. Initially, the system was to be implemented in 6 public facilities (1 county hospital and 5 primary care facilities) with a view to expanding to other facilities as resources became available. The county hospital had an existing IT system in place but was motivated to install an MOH-backed system to try to lower costs, improve system performance, and increase access to technical support.

**Table 4:** Levels of care defined by the Kenya Health Policy 2014-2030.

Level of Care	Facilities
Level 1: Community	Community: Village/households/families/individuals
Level 2: Primary care facilities	Dispensaries or clinics and health centers
Level 3: County hospitals	Primary care hospitals; secondary care hospitals
Level 4: National referral hospitals	Tertiary care hospitals

To summarize the implementation of the AfyaEHMS project, we use a framework presented by Jawhari et al [30]. Their synthesis of key messages appearing in literature present a framework that can be used to summarize the benefits and barriers to EMR implementation in developing countries as systems, people, process, and products. Systems relates to infrastructure available such as power and a reliable network, people relates to factors to do with users such as their training and attitudes, process relates to how the system is implemented, for example, the change management process and time of deployment, while product relates to the system itself and how it interoperates with other applications. Table 5 summarizes the implementation of versions 1 and 2 of AfyaEHMS project.

### Way Forward

Following the experiences during version 1 and 2 system implementations, the project implementers identified challenges and proposed solutions as outlined in Table 6.

The wide scope of the project was a major challenge during system development and implementation of Versions 1 and 2. The scope of the system was thus scaled back from a mix of health centers and county hospitals to cover only primary care facilities (Level 2) for the time-being. This allowed for faster rollout to more sites. Once this was done and at a stable level, then scale up to larger hospitals would be considered. More counties have since been targeted for rollout; currently 5 counties (Baringo, Kilifi, Bungoma, Garissa, Turkana) are on board with more being targeted for rollout with over 70 health centers having been installed to date. Health centers provide a wide range of predominantly outpatient services, such as basic curative and preventive services for adults and children, as well as reproductive health services minor surgical services and are staffed by midwives or nurses, clinical officers, and occasionally by doctors. They augment their service coverage with outreach services and refer severe and complicated conditions to the appropriate level, such as the district hospital [31].

Scaling back the system implementation to only health centers allowed the developer to focus their efforts on system modules other than the finance module. The finance module was an important module to large hospitals that collect revenue but not to health centers where care is free and was a barrier to full system implementation in Versions 1 and 2. Modules that are in use at the health centers include: patient registration, a clinical module for general outpatient services, pharmacy, laboratory, and a maternity module to cover antenatal services and the MCH clinic. Currently, the EHR does not cover the comprehensive care clinics (CCC), that give HIV care, but discussions are underway to find ways of integrating with existing systems and including the CCC functionality in a future version. Other key developments have been the implementation of a reporting module that generates a file that can be uploaded to DHIS2 (the national reporting system). There are plans to introduce internet to the facilities, and this will facilitate automatic reporting of data to DHIS2.

A Kenyan software development company has been engaged to develop the new system which should allow for faster system development and quicker resolution of emerging issues. A plan is in place to have a support team that will be responsible for the system handover over a longer period (6 months) allowing them to provide better system support to the health centers and thus ensuring system sustainability. To further facilitate faster resolution of issues, the project manager uses WhatsApp groups to support implementations within the counties whose membership includes system users and facility incharges.

Early stakeholder engagement with new counties helped to foster a feeling of ownership which was a major barrier to system adoption during the previous installation. The new county administration teams have in turn been supportive of the system implementation by availing resources (monetary and staffing) when necessary. Additionally, during implementation, the project implementation team now trains Trainers of Trainers; a team comprising a national team member, health workers (eg, health records information officer, pharmacist, lab technologist, or nurse) who have worked within the county, and members with IT training. These teams undergo a 3-day training supported by funds from the county and WHO. The eHealth unit at the MOH also sends a member to be present each time an implementation is taking place.

Previously, there were health centers that did not have electricity for up to 2 weeks making system implementation and use impossible. For this implementation round, the use of solar power has been considered for some sites while in others, generators are in use; this is done at the start of the implementation at the site. Depending on the setup, if a generator was available then that would be used as backup in case of power outage, if it was not in a working condition, then efforts were made to fix it.

To counter the challenge of laptops posing a security concern due to theft, the project now employs the use of zero clients and a server. Zero clients are all-in-one computer terminals that occupy relatively less space and are easier to rollout and maintain. The network has also been setup using a Local Area Network as opposed to a wireless network, which was not reliable previously.

**Table 5:** System implementation—Versions 1 and 2.

Determinants	Version 1		Version 2 (demonstration by clerks)	
	Description	Challenges	Description	Challenges
Systems	Hardware: 15 laptops preloaded with Ubuntu Linux version 14.0 procured to be used in addition to the already existing hardware Networking: wired and wireless connections System setup to use laptops as client computers to access a central server allowing for portability Information technology (IT) staff (2) in charge of expanding the computer network and general troubleshooting of hardware issues Software support: provided by developer based in India	Workstations insufficient: approximately 30 to 35 computers needed to cover all the departments Laptops raised concerns of theft leading to delay in deployment of equipment in some sites Inadequacies in infrastructure such as weak or missing Wi-Fi signal and poor 3G network made connecting to the internet difficult Lack of electric power in a site leading to delay in deployment Resolution of software issues were perceived to take too long	Network improved ensuring accessibility from any computer connected to the network Additional staffing in IT department (3 staff and 4 interns)	Only the developer team could make software modifications to the system
People	September 2014: training on system use completed at 4 (1 level-5 hospital and 3 level-3 facilities) out of 6 target facilities concurrently Training completed at site of work IT staff trained on system installation on the server	Low levels of computer literacy Reported high user workload Limited support staff Lack of user buy-in	Some staff members trained on system use though this was not done for all staff The data clerks were also trained and expected to train other users such as nurses on system use	A major barrier to training all the staff was that the schedules for the different staff would not allow for all of them to be gathered at one place Lack of user buy-in to the project as the development team and end-users were in different countries and had only limited time for communication and training
Process	Use of data clerks to enter data from physical patient files to counter shortage of staff and busy work schedules		Shifted responsibility of data accuracy and integrity to clerks, a role normally assigned to nurses and clinicians in order to verify the data	Commissioning of a major project resulted in a shift of attention and resources hence not feasible to give the required attention and resources to the Afya Electronic Health Management System (in short AfyaEHMS) deployment
Products	Modules: patient registration, outpatient, inpatient, laboratory, pharmacy, health records and hospital inventory	Request for additional functionality (more comprehensive symptom lists, an option to enter free text) Need for a more user-friendly International Statistical Classification of Diseases and Related Health Problems 10th Revision code list for diagnosis Need to reduce number of steps required to achieve tasks (eg, pharmacy and inventory modules) Patient identification number generated by the system was too long Finance module not as comprehensive as the preexisting system	Modules updated to incorporate requested changes	Comprehensive testing needed to ascertain whether all changes requested were captured

**Table 6:** Challenges and proposed solutions.

Challenge	Proposed solution
Poor support and use of external developers	Need for a longer-term support solution
	Need for local developers to get involved sooner rather than later in the project
Poor support from county management	Engage with all stakeholders from an early stage to foster system ownership and ensure they are consulted during development and implementation
Wide project scope	Scale down system to cover smaller health facilities
Infrastructure issues	Better hardware solutions needed to ensure easier overall maintenance.

## Discussion

### Principal Findings

This case study describes a novel idea: to develop and deploy an EHR using existing open source software for use in public health facilities in Kenya. The project implementation was faced with some of the common problems with EHR roll-outs in both low-income settings, where EHRs have generally been used in smaller clinics and in high-income settings, where EHR implementations have been attempted with varying degrees of success in larger hospitals.

In common with other low-resource eHealth projects, the lack of power, inadequate hardware, and networking were a major challenge to system setup during the deployment of Version 1 and 2. In earlier projects, multiple power sources have been used to ensure the availability of power and system availability if one of the sources fails [32,33]. For this project, the implementing team addressed the power and hardware issues by adding extra local human resources for troubleshooting and fixing issues as they arose.

There is growing consensus in the international eHealth literature that overcoming challenges that are due to human factors such as computer literacy and attitudes can be a major step toward successful EHR implementation in both developed and developing countries [30,32,34,35]. We found that issues due to human factors caused significant problems with the implementations we studied with concerns about user acceptance of the new system. The users felt that the system belonged to *outsiders*, and this affected the system ownership. To overcome this, using system design strategies that are more inclusive, such as codesign or participatory design, at an early stage can be employed to help ensure system buy in from potential users [35,36]. In a similar case study implementing an EMR system at a large hospital, management of different users’ expectations was noted as an important aspect of the successful implementation [35]. Different stakeholders have different interests and abilities to influence the process, which needs to be managed and planned for at an early stage of system implementation [37]. This coupled with managing the scope of the system using, for example, Agile software development principles [38] could help in gradually developing a system until it is fully operational while keeping relevant stakeholders on-board.

Hospitals are complex organizations [39] and, as such, implementing any new technology requires careful planning and management. The literature shows that eHealth implementers should take into account the existing workflows and organizational culture to come up with a change management plan that takes into account the different actors and their views [40]. Large hospitals operate with highly hierarchical structures and varying levels of availability of staff and these factors need to be considered to ensure successful implementation. Scaling back the implementation to the primary care facilities, which are less complex, has enabled the implementers to better deploy a better working system with plans to build on it once system operations stabilize.

Historically, data clerks or scribes have been used to enter clinical data into EHRs both in low-income [41,42] and high-income countries [43] in order to overcome the challenge of high clinician workload while deploying an EHR. The HIV clinics that use EHRs in Kenya have used data clerks through external support. However, for developing countries that are resource constrained, the use of data clerks on a long-term basis needs to be explored to establish its sustainability. The use of structured forms has been shown to improve the quality of documentation [44,45], a step toward improved quality of care. It has also been associated with increased generation of useful data [30] in comparison to the use of unstructured forms that rely on free-text input.

From an early stage in this case study, the implementers envisioned that system support would be offered through a help desk, where general system issues are addressed, and a community of practice (COP) where users could share experiences and learn from one another. COPs are often used in EHR implementations to provide an avenue to share innovations, help foster higher system utilization through mentor support, allow new staff members to quickly find clinical staff that are more familiar with the system, and provide an avenue to develop standardized templates for use in practice. They can also allow users greater influence in issues such as coordinating support with the vendor to optimize feature requests and training [46]. While some COPs may be self-organizing, the AfyaEHMS COP would have benefited greatly from a facilitator or coordinator. A dedicated facilitator helps the community to focus on its domain, maintain relationships, and develop its practice [47].

Use of open source software may offer some respite from the high costs of proprietary software, which is a well-documented barrier to adoption of EHRs [48]. Open source software is also often associated with online supporting communities that are constantly improving the software. The Esaude community is an example of a local community focused on the development and implementation of a Mozambican specific configuration of the OpenMRS medical record software and the integration into a national eHealth architecture [49]. Members of the community collaborate and participate in the global OpenMRS community where they learn from the collaboration model and receive mentorship for learning and developing the software. Tapping into these communities may help reduce over reliance on one individual or software vendor for system updates, which are needed as the software evolves to suit the changing needs of the users and contributes to the principle of operational self-sufficiency noted by Surana et al [50] as being key to implementing any information and communication technology project. Such a community would bring on-board as many interested parties as possible that can continue to contribute to the project. Additionally, partnering with higher learning institutions may be a useful way to get more technical input into the project by engaging students to rapidly develop sections or modules of the system that might need improvement through boot camps or as part of their coursework through projects. An example of where this has been implemented is Rwanda, where a training program for local computer science graduates is being run to enable them to contribute to the implementation of the national EMR system [51].

### Conclusions

Implementing EHR systems is a challenging process in high-income settings. In low-income settings, such as Kenya, open source software may offer some respite from the high costs of software licensing, but the familiar challenges of clinical and administration buy-in, the need to adequately train users, and the need for the provision of ongoing technical support are common across the North-South divide. We hope this case study will provide some lessons and guidance for other challenging implementations of EHR systems as they continue across Africa.

## Abbreviations

AfyaEHMS: Afya Electronic Health Management System
CCC: comprehensive care clinics
CDC: Centers for Disease Control and Prevention
COP: community of practice
DHIS2: District Health Information System Version 2
EHR: electronic health record
EMR: electronic medical record
ESG: EMR Standards and Guidelines
HIE: health information exchange
HMIS: Health Management Information Systems
IQCare: International Quality Care
IT: information technology
ITECH: International Training and Education Center for Health
KEMRI: Kenya Medical Research Institute
MOH: Ministry of Health
NASCOP: National AIDS and STI Control Programme
OpenMRS: Open Medical Record System
SDMX.HD: Statistical Data and Metadata eXchange for the Health Domain
TB: tuberculosis
WHO: World Health Organization
